# MicroRNA-652 induces NED in LNCaP and EMT in PC3 prostate cancer cells

**DOI:** 10.18632/oncotarget.24937

**Published:** 2018-04-10

**Authors:** Robert K. Nam, Tania Benatar, Yutaka Amemiya, Christopher J.D. Wallis, Joan Miguel Romero, Melina Tsagaris, Christopher Sherman, Linda Sugar, Arun Seth

**Affiliations:** ^1^ Division of Urology, Sunnybrook Health Sciences Centre, Sunnybrook Research Institute, University of Toronto, Toronto, ON, Canada; ^2^ Platform Biological Sciences, Sunnybrook Research Institute, Toronto, ON, Canada; ^3^ Genomics Facility, Sunnybrook Research Institute, Toronto, ON, Canada; ^4^ Department of Anatomic Pathology, Sunnybrook Health Sciences Centre, Toronto, ON, Canada; ^5^ Department of Laboratory Medicine and Pathobiology, University of Toronto, Toronto, ON, Canada

**Keywords:** miR-652-3p, miRNA, prostate cancer, NED, EMT

## Abstract

MicroRNAs (miRNAs) are small noncoding RNA molecules that post-transcriptionally regulate gene expression. Dysregulation of miRNAs is frequently associated with disease and, in particular, is involved in prostate cancer progression. Next generation miRNA sequencing identified a panel of five miRNAs associated with prostate cancer recurrence and metastasis. High expression of one of these five miRNAs, miR-652, correlated significantly with an increased rate of prostate cancer biochemical recurrence. Overexpression of miR-652 in prostate cancer cells, PC3 and LNCaP, resulted in increased growth, migration and invasion. Prostate cancer cell xenografts overexpressing miR-652 showed increased tumorigenicity and metastases. We found that miR-652 directly targets the B” regulatory subunit, PPP2R3A, of the tumor suppressor PP2A, inducing epithelial-mesenchymal transition (EMT) in PC3 cells and neuroendocrine-like differentiation (NED) in LNCaP cells. The mesenchymal marker N-cadherin increased and epithelial marker E-cadherin decreased in PC3 cells overexpressing miR-652. In LNCaP cells and xenografted tumors, overexpression of miR-652 increased markers of NED, including chromogranin A, neuron specific enolase, and synaptophysin. MiR-652 may contribute to prostate tumor progression by promoting NED through decreased PP2A function. MiR-652 expression could serve as a biomarker for aggressive prostate cancer, as well as provide an opportunity for novel therapy in prostate cancer.

## INTRODUCTION

Hormone refractory prostate cancer is often associated with neuroendocrine differentiation (NED) [1]. Tumors with increased NED are more aggressive and correlate with poor prognosis [2–4]. NED in prostate cancer has been associated with specific signaling pathways. Constitutive activation of AKT promotes NED, and NED induced by androgen withdrawal is suppressed by the phosphoinositol-3-kinase (PI3K)/AKT inhibitors [5, 6]. Several studies suggest that activation of the Wnt pathway promotes NED of prostate cancer cells [7, 8]. Wnt expression is elevated in hormone-independent prostate cancer [9]. β-catenin, a component of the Wnt signaling pathway, has been found to induce NED when overexpressed in LNCaP cells [8]. Sustained extracellular signal-regulated kinase (ERK)-1/2 activation is proposed as one of the major mechanisms in NED of prostate cancer cells, playing a critical role in converging of multiple signaling pathways for NED [10]. Upregulated activity of the Ras-Raf-mitogen-activated protein (MEK)/ERK pathway is associated with prostate cancer progression and poor prognosis, correlating with increased tumor grade of primary or metastatic prostate cancer and tumor relapse after therapy [11–14]. AR (androgen receptor) is downregulated upon NED of LNCaP cells [15], and in NE (neuroendocrine) cells in malignant prostatic tissues [16]. ERK-1/2 and AKT activation correlate with loss of AR upon androgen deprivation, and is sufficient to regulate AR [15, 17].

Most prostate adenocarcinomas contain some scattered NE cells, which are kept quiescent through a strong growth inhibitory signal mediated by the IL8-CXCR2-p53 pathway [18, 19]. Mutation of p53, likely a result of environmental pressure from hormonal therapy, inactivates this pathway, leading to hyperproliferation and aggressive behavior of NE cells, resulting in NED characterized as small cell neuroendocrine carcinoma (SCNC) [18, 20].

Specific miRNAs have been linked to NED of prostate cancer. Hypoxia has been shown to promote NED of prostate cancer cells by inducing the miR-106b∼25 cluster which comprises miR-106b, miR-93 and miR-25 [21]. MiR-221 overexpression induced NED in LNCaP cells, and was also increased in the plasma of prostate cancer patients versus controls [22]. MiR-663 overexpression has also been shown to induce NED in LNCaP cells, and expression profiling indicates high miR-663 expression in castration-resistant prostate cancer (CRPC) patients with associated poor prognosis [23].

We recently identified miRNA-based biomarkers that correlate with recurrent and metastatic prostate cancer following radical prostatectomy [24]. Next generation miRNA sequencing identified 33 miRNAs associated with prostate cancer metastasis. A panel of five miRNAs were selected, miRs-301a, 652, 454, 223 and 139 which strongly predicted metastasis (AUC = 95.3%, 95%C.I.:84%–99%) [24]. Three of these miRNAs were overexpressed in prostate tumors, one of which was miR-652. Functional investigation of overexpressed miRNAs was prioritized due to their greater potential for use as diagnostic markers and therapeutics.

Biological functions of miRs-301a and 454 in prostate cancer have been reported previously [24]. This study examined the biological mechanism by which miR-652 increases prostate cancer aggressiveness and NED. We identified the B” regulatory subunit, PPP2R3A, of the tumor suppressor serine/threonine protein phosphatase 2A (PP2A), as a potential target of miR-652 using the miRNA target prediction programs miRanda and PicTar. PP2A is a tumor suppressor that is essential in the regulation of major signalling pathways which can contribute to carcinogenesis [25]. PP2A is a negative regulator of many signaling pathways promoting cell growth, proliferation, and survival, such as PI3K/AKT/mTOR, Wnt/β-catenin, c-Myc and the MAP/ERK family of kinases [26–29]. Decreased activity of PP2A is a recurrent observation in many types of cancers, such as colorectal cancer and breast cancer [30, 31]. Interestingly, when the frequency of PP2A subunit mutations were examined across 9759 tumor samples, PPP2R3A had the second highest mutational rate (12.16 %) overall, and the highest amongst the 11 B regulatory subunits genes [32].

## RESULTS

### MiR-652 expression correlates with increased patient prostate cancer recurrence but not metastasis

To determine whether miR-652 could be used as an independent prognostic marker for patients with localized prostate cancer, we examined a cohort of patients who underwent surgery for prostate cancer who were separate and independent from the initial Discovery Set. Study subjects were selected from a cohort of 654 patients who underwent radical prostatectomy for clinically-localized prostate cancer between 1990 and 2000 at a single, tertiary care centre (Sunnybrook Health Sciences Centre). Patients were excluded if they had a history of other non-melanoma cancer (*n* = 7), incomplete medical chart information (*n* = 15), incomplete pathology tumour information (*n* = 9), or had neoadjuvant hormone therapy (*n* = 14). The endpoints of interest were (1) the development of biochemical recurrence based on serum prostate specific antigen (PSA) levels and (2) the development of bone or visceral metastasis, based on medical imaging evaluation.

Of the 609 patients who were included in the study (and who were not part of the Discovery Set), qPCR measurements of the miRNAs could not be obtained on 24 tumour samples leaving 585 (96.1%) patients for final analysis. Of the 585 patients, 197 (33.7%) developed biochemical recurrence, and 32 (5.5%) developed metastasis, after a median follow-up of 8.4 years (interquartile range (IQR): 4.6–10.7 years). The median age at the time of surgery was 62.8 years (IQR 57.2–67.1 years), the median PSA was 6.5 ng/mL at the time of diagnosis (IQR 4.4–9.5 ng/mL), and the majority of patients (61.2%, *n* = 300) had cancer confined within the prostate. The majority of patients had Gleason Score 7 tumours (62.2%, *n* = 364). Stage, grade, PSA level at diagnosis, positive surgical margins, and lymph node involvement were important prognostic factors for recurrence and metastasis [33].

**Figure 1:**
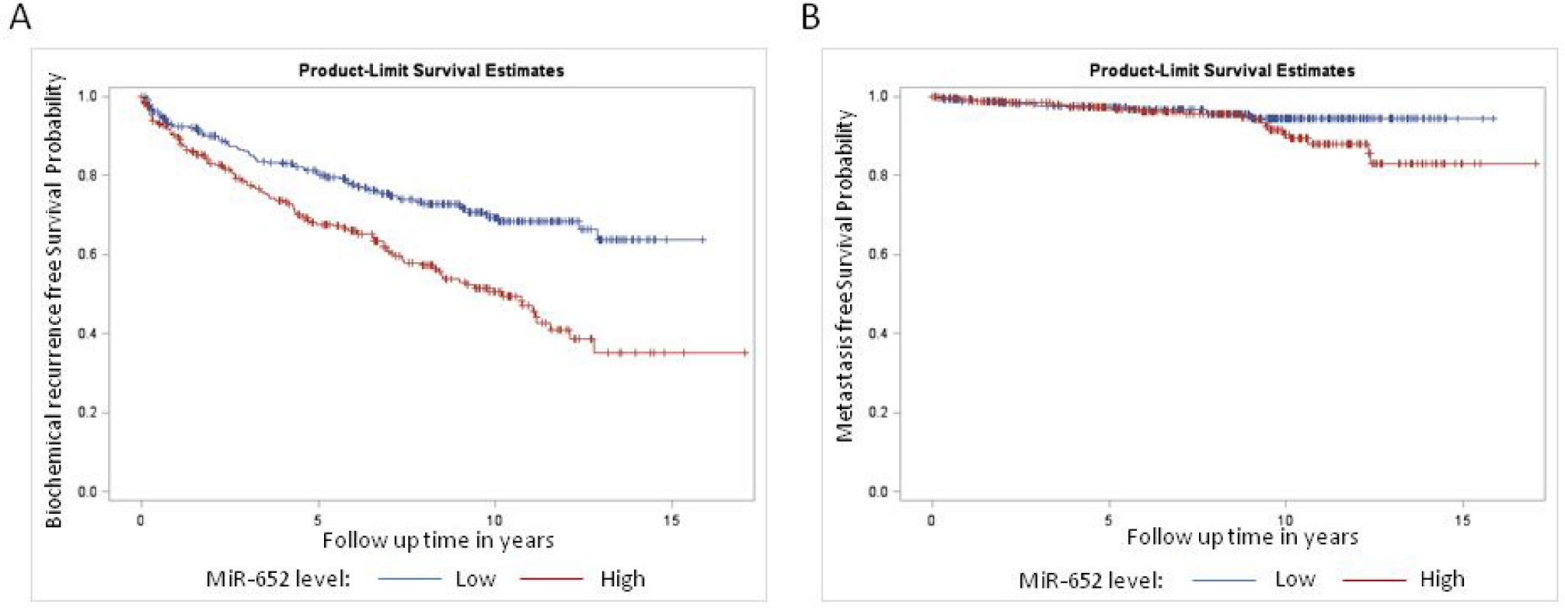
Kaplan Meier survival analysis for (**A**) biochemical recurrence and (**B**) metastasis among patients treated for clinically localized prostate cancer, stratified by miR-652 expression level. There was a significant difference in survival among the miR-652 expression groups for biochemical recurrence (*P* < 0.0001) but not metastasis (*p* = 0.10).

The median miR-652 expression level was significantly higher among patients who developed recurrence compared to patients who did not (0.239 versus 0.207, *p* < 0.0001) and among those patients who developed metastasis compared to those who did not (0.247 vs. 0.215, *p* = 0.0277). To evaluate the clinical significance of the miR-652 expression, we grouped patients into those with a low and high risk based on their miR-652 levels, with a cut-off defined by the median value of the miR-652 distribution (0.216). Patients with a low miR-652 score had significantly decreased risk of biochemical recurrence (*p* < 0.0001), but not metastasis (*p* = 0.1038; Figure 1A and 1B).

MiR-652 expression category was associated with known prognostic factors including PSA level, grade and pathologic stage (Table 1). After adjusting for patient age, grade, stage, PSA level, margin status and lymph node involvement, patients with a high miR-652 score had a higher rate of biochemical recurrence (HR = 1.47, 95% C.I.: 1.09–1.98, *p* = 0.0125; Table 2). On similar multivariable analysis, there was no association between miR-652 level and metastasis (HR = 1.16, 95% C.I.: 0.54–2.48, *p* = 0.71; Table 2) compared to those with a low score. However, conclusions regarding the association between miR-652 expression and metastasis are limited due to the small number of patients who developed metastases.

**Table 1:** Baseline characteristics of study cohort

	No recurrence (*n* = 388)	Recurrence/metastasis (*n* = 197)	*p*-value
Age (median, IQR)	62.12 (56.7–66.6)	63.31 (58.4–68.2)	0.0138
PSA (ng/mL; median, IQR)	6.00 (4.0–8.7)	7.30 (5.3–10.5)	<0.0001
PSA (ng/mL)			<0.0001
≤4	98 (25.3)	24 (12.2)	
4–10	215 (55.4)	110 (55.8)	
>10	75 (19.3)	63 (32.0)	
Grade (Gleason score)			<0.0001
5–6	126 (32.5)	12 (6.1)	
7	245 (63.1)	147 (74.6)	
8–10	17 (4.4)	38 (19.3)	
Pathologic Stage			<0.0001
Organ Confined (pT2)	296 (76.3)	68 (34.5)	
Extraprostatic Extension (pT3a)	78 (20.1)	82 (41.6)	
Seminal Vesicle Involvement (pT3b)	14 (3.6)	46 (23.4)	
Adjacent Organ Invasion (pT4)	0 (0)	1 (0.5)	
Margin positivity	116 (29.9)	113 (57.4)	<0.0001
Lymph node status			<0.0001
Negative	105 (27.1)	80 (40.6)	
Positive	2 (0.5)	12 (6.1)	
Missing	281 (72.4)	105 (53.3)	

For age and PSA as a continuous variable, *p*-values were calculated using the Wilcoxon rank sum test.For the rest of the variables, *p*-values were calculated using the Chi-square test.

**Table 2:** Univariate and multivariate Cox proportional hazard modeling of prognostic factors for prostate cancer recurrence and metastasis

Prognostic Factor	Univariate Crude Hazard Ratio (95% C.I.)	*p*–value	Multivariate Adjusted Hazard Ratio (95% C.I.)	*p*–value
Biochemical Recurrence				
Age	1.00 (0.983–1.02)	0.9800	0.998 (0.985–1.01)	0.7227
MiR-652 Level				
Low	1.0		1.0	
High	1.926 (1.44–2.57)	<0.0001	1.466 (1.09–1.98)	0.0125
Histologic Grade (Gleason Score)				
5–6	1.0		1.0	
7	5.897 (3.26–10.65)	<0.0001	3.537 (1.92–6.51)	<0.0001
8–10	17.91 (9.28–34.57)	<0.0001	5.887 (2.91–11.92)	<0.0001
Pathologic Stage				
Organ Confined (pT2)	1.0		1.0	
Extraprostatic Extension (pT3a)	3.721 (2.69–5.15)	<0.0001	2.406 (1.72–3.37)	<0.0001
Seminal Vesicle Involvement (pT3b)	8.20 (5.58–12.03)	<0.0001	3.820 (2.42–6.04)	<0.0001
Adjacent Organ Invasion (pT4)	10.33 (1.43–74.78)	0.0207	3.783 (0.495–28.92)	0.1998
PSA (ng/mL)				
≤4	1.0		1.0	
4–10	2.265 (1.45–3.54)	0.0003	1.722 (1.09–2.71)	0.0190
>10	2.954 (1.84–4.74)	<0.0001	1.119 (0.673–1.86)	0.6638
Margin status				
Negative	1.0		1.0	
Positive	2.774 (2.09–3.69)	<0.0001	1.962 (1.46–2.64)	<0.0001
Lymph node status				
Negative	1.0		1.0	
Positive	3.504 (1.90–6.46)	<0.0001	1.967 (1.02–3.79)	0.0029
Missing	0.471 (0.351–0.631)	<0.0001	0.625 (0.458–0.851)	0.0430
Metastasis				
MiR-652 Level				
Low	1.0		1.0	
High	1.80 (0.878–3.68)	0.1087	1.156 (0.538–2.48)	0.7102
^*^Histologic Grade (Gleason Score)				
5–7	1.0		1.0	
8–10	13.00 (6.48–26.07)	<0.0001	7.81 (3.44–17.73)	<0.0001
^*^Pathologic Stage				
pT2 or pT3a	1.0		1.0	
pT3b or pT4	7.423 (3.65–15.09)	<0.0001	2.36 (0.96–5.78)	0.0614
^*^PSA (ng/mL)				
≤10	1.0		1.0	
>10	2.984 (1.49–5.97)	0.0020	1.473 (0.684–3.17)	0.3227

*Groups collapsed from three categories into two due to sample size limitations among patients who developed metastasis.

### MiR-652 overexpression increases prostate cancer cell growth

**Figure 2:**
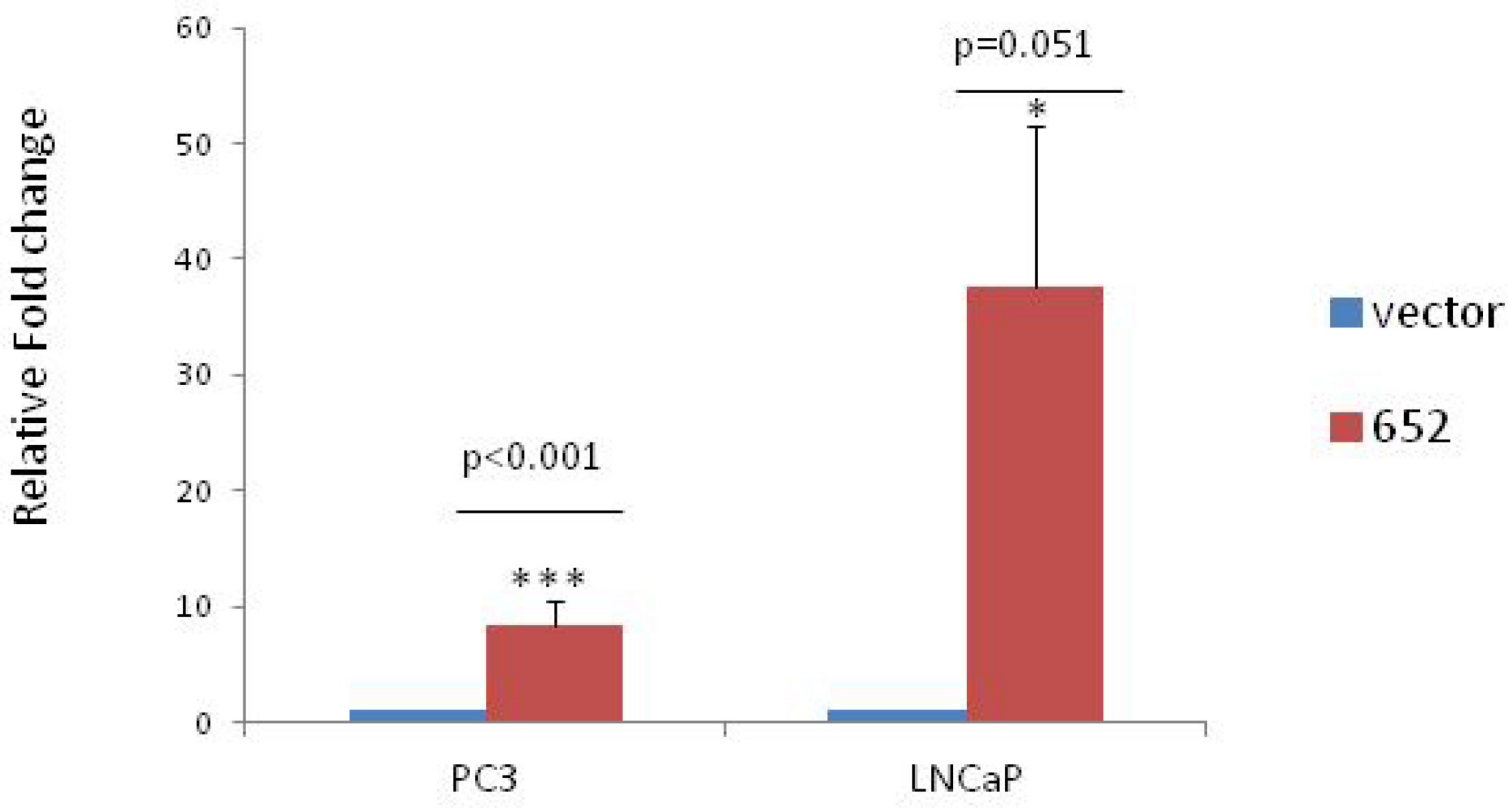
Stable overexpression of miR-652 in PC3 and LNCaP prostate cancer cell lines Quantitative PCR of miR-652 expression was performed on 1000 ng of PC3 and LNCaP cells stably transfected with either empty vector or miR-652 containing plasmids. Output is shown as relative fold change normalized to vector control in each group (1). At least 3 separate groups were analyzed per cell line. Mir-652 was normalized to endogenous control miR-28.

Overexpression of mir-652 in PC3 and LNCaP cells was done using the PCMV-MIR plasmid containing human miR-652 or empty vector control. Quantitative PCR (qPCR) after selection found that PC3 lines had 8.3 fold and LNCaP had 37.6 fold more miR-652 relative to empty vector transfected controls (Figure 2).

**Figure 3:**
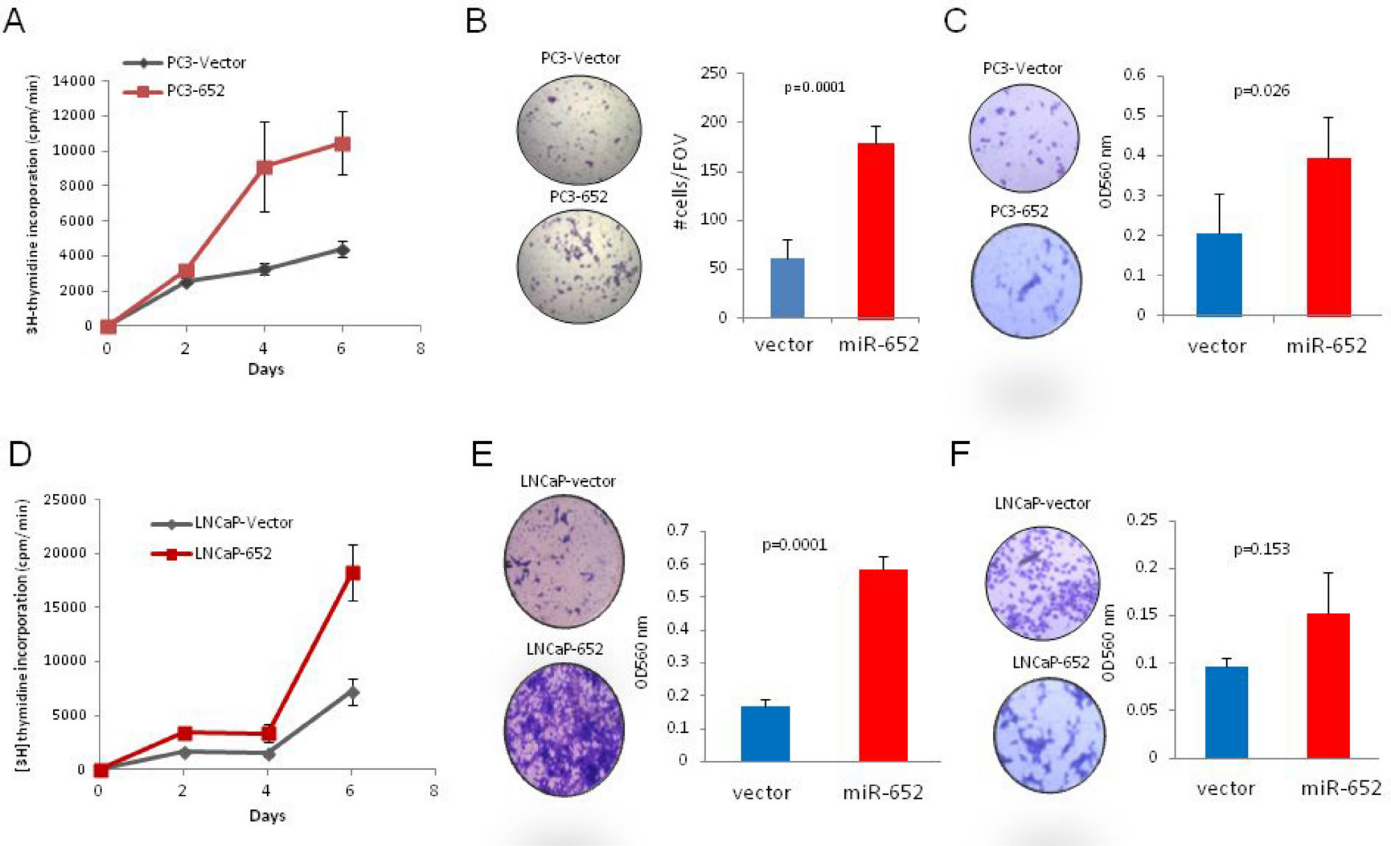
MiR-652 overexpression promoted PC3 and LNCaP prostate cancer cell growth migration and invasion (**A** and **D**) For proliferation, cells were plated in triplicate, and radioactive incorporation was represented graphically. Experiments were performed in triplicate for overexpressing miR-652 PC3 cells (PC3-652) and vector control cells (PC3-vector) (A) and LNCaP (LNCaP-652) and vector control cells (LNCaP-vector) (D). (**B** and **E**) Migration of cells was assayed after 72 hr. Overexpressing miR-652 PC3 cells (B) or miR-652 LNCaP cells (E) were compared with empty vector control transfected cells. At least three separate fields of view (FOV) were counted per sample (B) or 3 triplicate wells were assayed by measuring OD 560 (E). Graphic representations are averages expressed with their standard deviation. *P* values were calculated by Student’s *T* test. (**C** and **F**) Invasiveness was performed, and assayed after 72 hr by measuring OD 560 nm of triplicate samples. Averages are shown with their SD error bars. Results are representative of at least three independent experiments.

MiR-652 overexpression increased the proliferative potential of PC3 and LNCaP cells compared to empty vector controls by 2.3 and 2.5 fold respectively (Figure 3A, 3D), cell migration was enhanced up to four-fold (Figure 3B, 3E) and invasiveness was increased only in PC3 cells (Figure 3C, 3F).

### PPP2R3A is a direct target of miR-652 in prostate cancer cells

**Figure 4:**
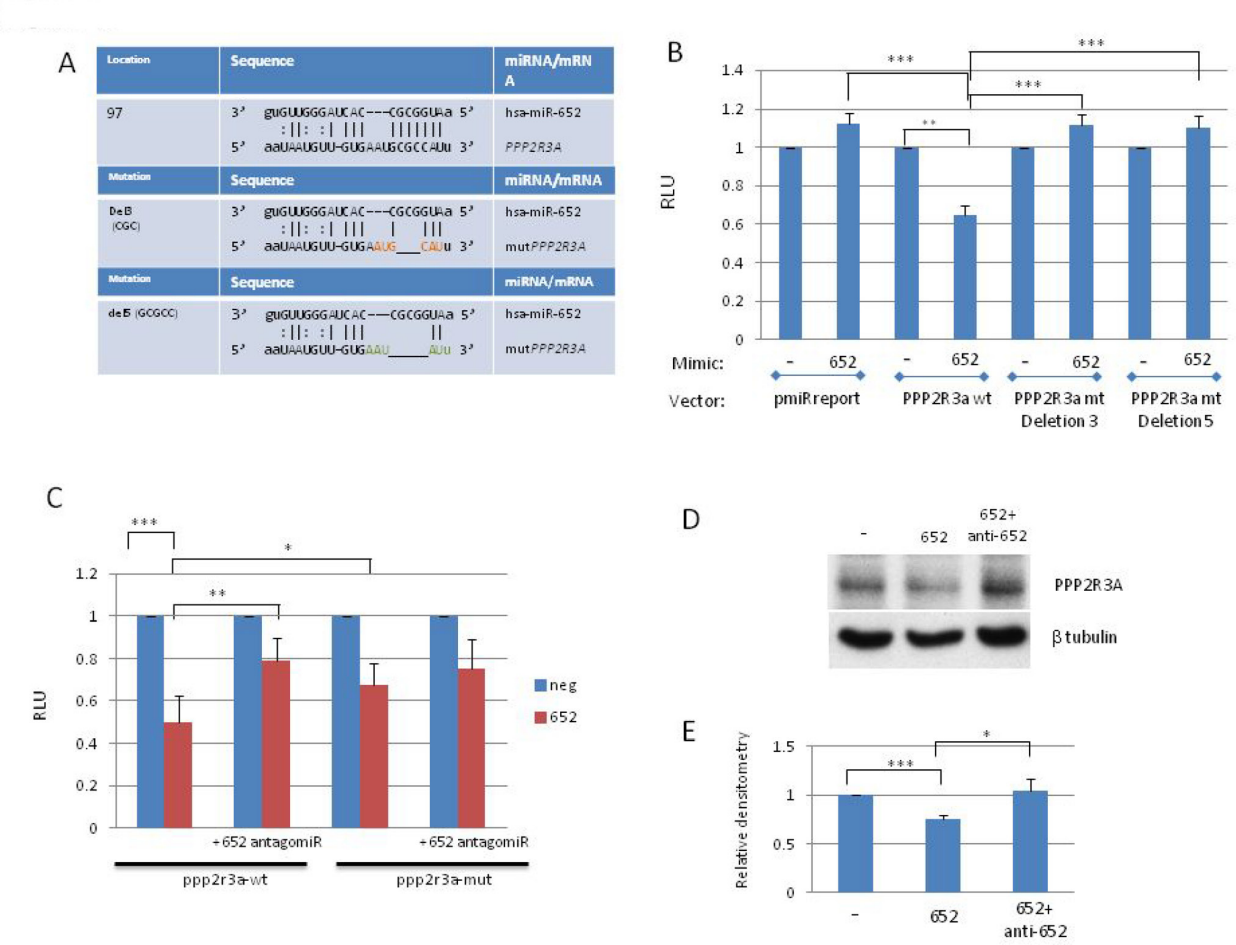
PPP2R3A is a direct target of MiR-652 (**A**) (top panel) Location of the miR-652 binding site within exon 2 (PR72) or exon 3 (PR130) of PPP2R3A isoforms. Second and third panels show deletions of either 3 bp (Del3) or 5 bp (Del5), respectively) created within the miR-652 binding site. (**B**) HEK293T cells were transfected with miR-652-3p mimics or negative control (NC) mimic, together with either empty pMIR-REPORT vector (pmiRreport), or pMIR-REP-PPP2R3A-Wt (PPP2R3A wt(wild type)) or PPP2R3A mt (mutant) deletion 3 or deletion 5 reporter plasmids along with pRL-TK. Luciferase activity was measured using a dual-luciferase reporter assay. The relative luciferase activity was calculated by LUC^firefly^/LUC^renilla^. The fold change of 652 mimic compared to NC mimic is shown; NC mimic set to 1. (**C**) HEK293T cells were transfected with either miR-652-3p mimics or NC alone, or with the addition of miR-652 antagomiR, together with either pMIR-REP-PPP2R3A-Wt (PPP2R3A wt or PPP2R3A mt deletion 3 or deletion 5 reporter plasmids along with pRL-TK. Luciferase assays were then performed as in (4B). (**D**) WB of PC3 cells transiently transfected for 3 to 7 days with either control mimic, miR-652 mimic, or miR-652 mimic together with miR-652 antagomir. Expression of PPP2R3A is shown, as is β-tubulin for protein loading control. (**E**) Densitometry of 3 independent WB performed as described in (4D), where PPP2R3A expression levels are normalized to β tubulin, and expressed as a percentage relative to cells transfected with negative control mimic. Data are represented as a mean ± standard error of the mean of three independent experiments and determined by the unpaired *t*-test (^*^P < 0.05; ^**^P < 0.01, ^***^P < 0.001).

The miRNA target prediction programs miRanda and PicTar identified PPP2R3A as a miR-652 target based on its strong miR-652 seed sequence (Fig 4A). PPP2R3A encodes the regulatory B” subunit of the serine/threonine phosphatase PP2A, an enzyme which regulates many cell growth associated pathways [34, 35]. The miR-652 binding site resides within exon 3 of the coding region of PPP2R3A, transcript variant 1(PR130) or exon 2 of transcript variant 2 (PR72). To confirm whether or not PPP2R3A is a direct target of miR-652, a dual luciferase reporter system was used. The intensity of fluorescence after miR-652 cotransfection was reduced significantly compared with the negative control (65% reduction) (Figure 4B). To verify that the reduced luciferase activity was a direct result of miR-652 binding to PPP2R3A, mutants of the miR-652 binding site were created by deleting 3 or 5 bp from the binding sequence (Figure 4A). In contrast to the wild type PPP2R3A sequence, no significant variation in luciferase activity was observed for either the empty reporter plasmid or 2 different deletion mutants of the miR-652 binding site of PPP2R3A (Figure 4B).

In order to confirm that PPP2R3A is targeted by miR-652, we have performed the rescue experiment. Addition of miR-652 antagomir significantly reduced miR-652 inhibition of luciferase activity from the PPP2R3A-luc construct. This is now shown by luciferase assay in Fig 4C. With miR-652 alone RLU was reduced to 49.5%. RLU was reduced to 79% when antagomir were included (*p* = 0.004). Deletion mutants of the miR-652 binding sites in the PPP2R3A UTR showed no significant differences in response to miR-652 alone or combined with miR-652 antagomir (*p* = 0.376) (Figure 4C).

To further confirm that PPP2R3A was a direct target of miR-652 in prostate cancer cells, PC3 cells were transfected with either miR-652 mimic alone, or in combination with anti-miR-652 antagomir, and compared to control cells transfected with a control mimic. Western Blotting showed that PPP2R3A expression is reduced significantly in the presence of miR-652 alone (Figure 4D–4E; *p* < 0.001). This reduction is abrogated upon addition of anti-miR-652 (Figure 4D–4E; *p* < 0.05), further verifying that PPP2R3A is indeed a direct target of miR-652 in prostate cancer cells.

### MiR-652 expression in prostate cancer cells induces EMT or neuroendocrine differentiation

**Figure 5:**
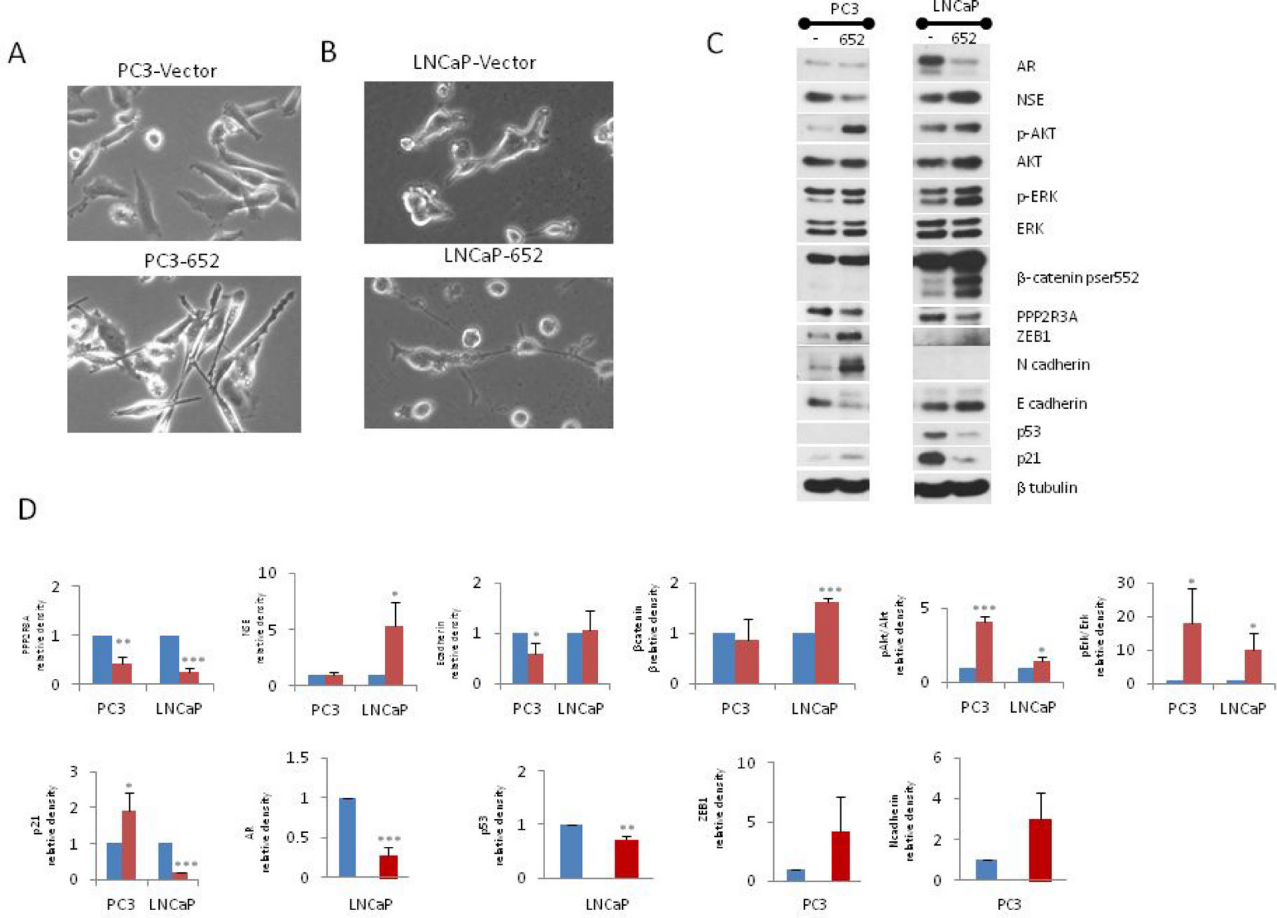
Transient transfection of miR-652 induces EMT in PC3, and NED in LNCaP prostate cancer cells PC3 and LNCaP cells were transfected with control mimic or miR-652 mimic for 13 days. (**A** and **B**) Monolayer morphology in phase contrast, magnification 20×. Top panel are cells transfected with control mimic, while bottom panel are transfected with miR-652 mimic. (**C**) Western blotting of total cellular proteins from miR-652 transfected or negative control mimic transfected PC3 (left panel) or LNCaP (right panel) cells. As an internal control, the level of β-tubulin expression is shown. (**D**) Densitometry of Western blots from 5C. Unless otherwise specified, β tubulin was used as internal control for each sample. Data are represented as a mean ± standard error of the mean of three independent experiments and determined by the unpaired *t*- test (^*^P < 0.05; ^**^P < 0.01, ^***^P < 0.001). Blue bars represent transfection with negative control mimic; Red bars represent transfection with miR-652.

Inhibition of PP2A activity increases growth and survival of androgen independent prostate cancer cells through a mechanism that involves AKT and ERK-1/2 signaling [36]. We tested the possibility that miR-652 engages a similar mechanism. PPP2R3A expression is indeed reduced in the presence of miR-652 mimic (Figure 5). Furthermore, these same cells had increased phospho-AKT, and phospho-ERK-1/2 as compared to PC3 cells transfected with negative control mimic. When PC3 cells are overexposed to miR-652 mimic, their morphology changes to a more elongated cell type, with oval shaped nuclei and several thin cytoplasmic processes, typical of mesenchymal cells (Figure 5A). The mesenchymal marker N-cadherin increased in the presence of miR-652 mimic, along with decreased E-cadherin and increased ZEB1 expression, indicating EMT switch (Figure 5C). These results suggested that miR-652 induces EMT when overexpressed in PC3 cells.

MiR-652 mimic also induced cellular morphological changes in LNCaP cells compared to negative controls, resulting in small round cell bodies together with long thin elongated cell processes resembling neurites (Figure 5B). This raised the possibility that in the presence of miR-652, LNCaP cells were undergoing NED. To explore this further, Western blotting was performed for markers of NED. We found that miR-652 induced the expression of neuron specific enolase (NSE), a marker of NED in LNCaP cells (Figure 5C), indicating differentiation to neuroendocrine pathway.

The loss of *TP53* is associated with development of prostatic SCNC [18]. We observed that p53 expression was significantly reduced in LNCaP cells (Figure 5C). Furthermore, p21 expression was also reduced in response to miR-652 expression in LNCaP cells. Downregulation of p21 in LNCaP cells treated with miR-652 is likely a consequence of p53 inhibition, since p53 can directly induces p21 expression [37–39].

The PR72 isoform of PPP2R3A was established as a novel negative regulator of the Wnt signaling pathway [40]. We therefore sought to determine whether downregulation by miR-652 of PR72 would result in activation of the Wnt pathway in prostate cancer. Activation of the Wnt signaling pathway has also been shown to promote NED, survival and migration of prostate cancer cells [7, 8]. Therefore we analyzed β-catenin expression, a measure of activated Wnt, by examining the activated form, phospho-β-catenin (Ser^552^), upon miR-652 overexpression in LNCaP cells. We found that after prolonged culture (13 days), phospho-β-catenin (Ser^552^) was significantly increased upon exposure of LNCaP cells to miR-652 (Figure 5C). Interestingly, addition of miR-652 did not increase phospho-β-catenin (Ser^552^) expression in PC3 cells, perhaps a necessary factor for NED.

### MiR-652 induces NED in xenografts

Overexpression of miR-652 increased *in vivo* tumor growth of xenografted PC3-652 and LNCaP-652 cells. Very small tumors grew from PC3-vector cells, while the PC3-652 injected cells produced tumors that were 10 fold larger than their PC3-vector control counterparts. No tumors grew from LNCaP-vector cells, while large tumors grew in all mice injected with LNCaP-652 cells. The LNCaP-652 tumors were dark red in colour which is typical of LNCaP xenografts and has been shown to be due to extensive neovascularisation [41, 42].

**Figure 6:**
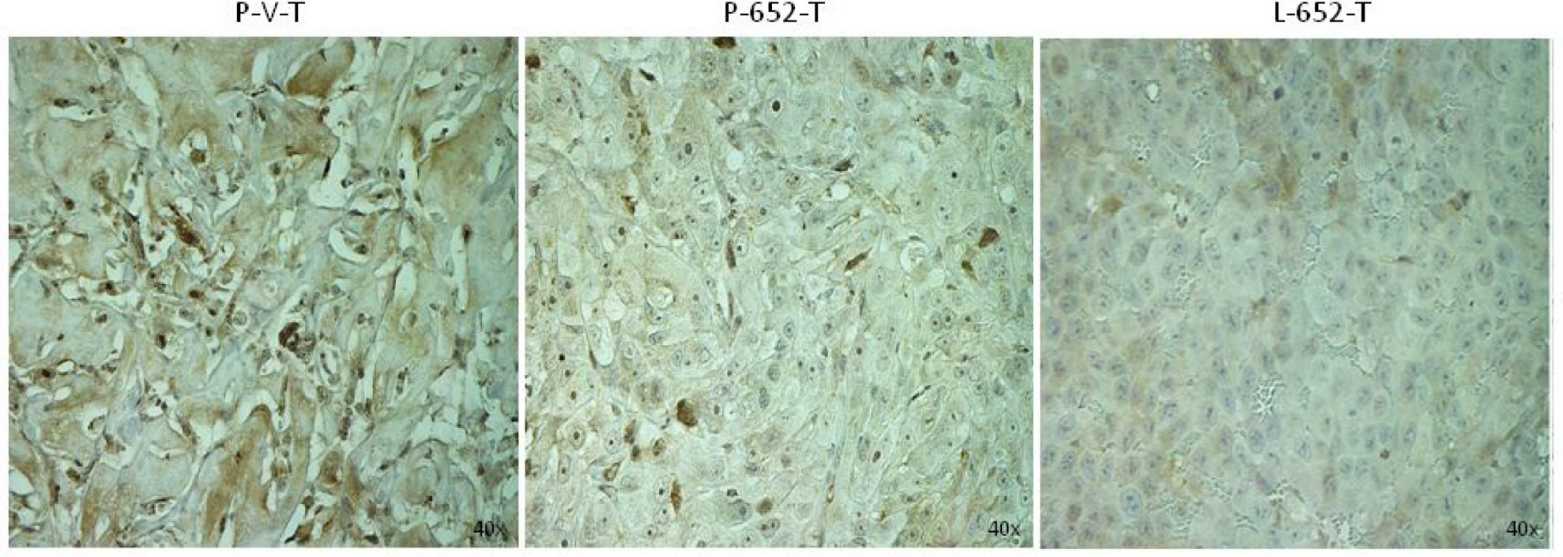
MiR-652 expressing xenografts have reduced levels of PPP2R3A Sections of xenografted tumors from NOD/SCID mice injected with PC3 or LNCaP cells overexpressing miR-652 or vector control as indicated. Sections were examined by IHC for PPP2R3A expression as indicated. Samples are PC3-vector (P-V-T), PC3-652 (P-652-T), or LNCaP-652 xenografts (L-652-T). Magnifications are 40×.

To confirm that miR-652 inhibits PPP2R3A *in vivo*, we examined xenografts derived from miR-652 overexpressing PC3 and LNCaP cells for PPP2R3A expression. We found that miR-652 inhibited the expression of PPP2R3A protein in xenografted tumors generated from both PC3-652 and LNCaP-652 expressing cell-lines compared to xenografts produced from empty vector (Figure 6).

**Figure 7:**
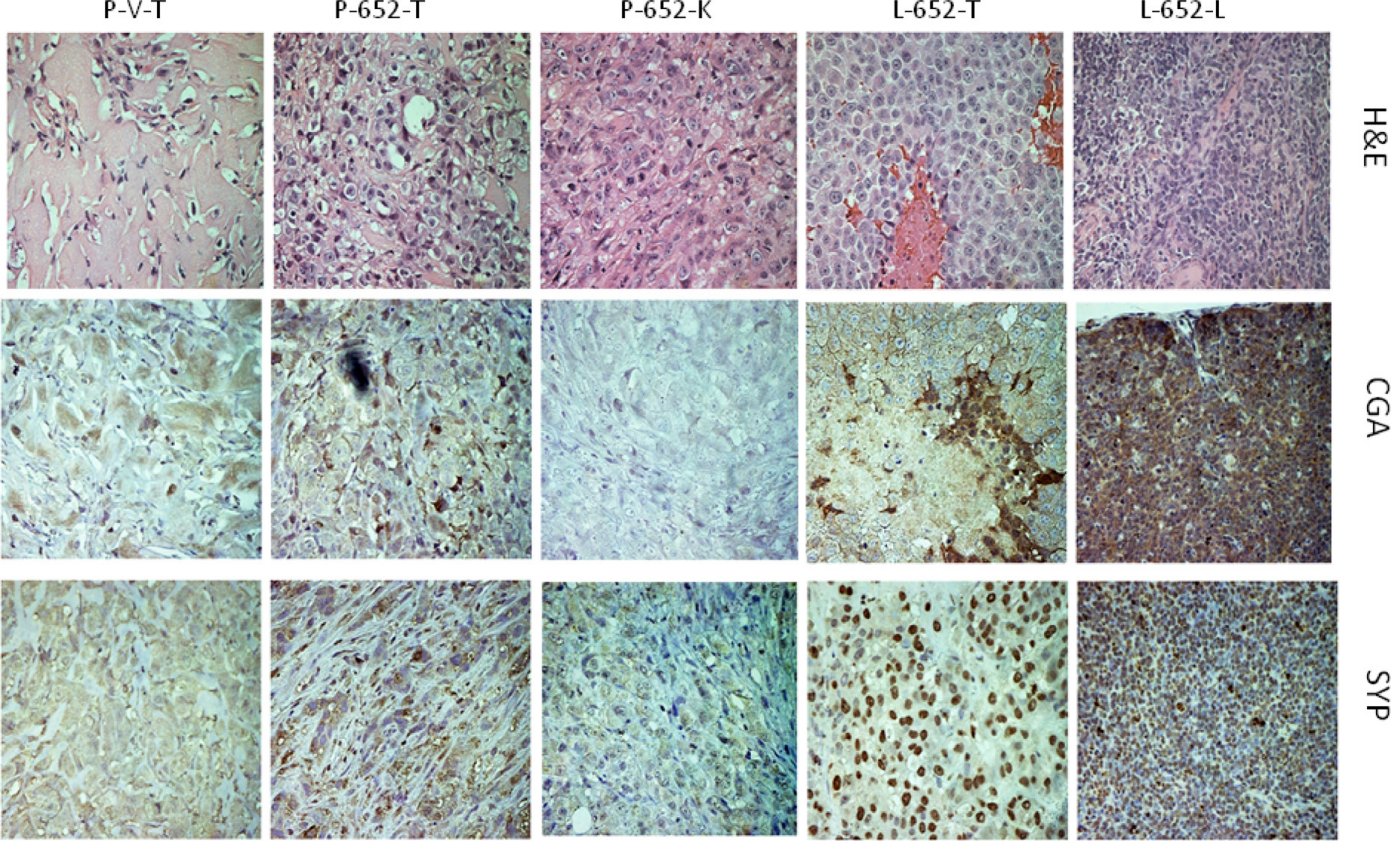
Expression of CGA and SYP in xenografted tumors and metastases from PC3-652 and LNCaP-652 cells H&E, Chromogranin A (CGA) and Synaptophysin (SYP) staining of sections of xenografted primary tumors and kidney or lung metastasis derived from NOD/SCID mice injected with either PC3 or LNCaP cells overexpressing miR-652 or vector controls as indicated. Samples are PC3-vector primary tumors (P-V-T), PC3-652 primary tumors (P-652-T), PC3-652 kidney metastasis (P-652-K), LNCaP-652 primary tumor (L-652-T) and LNCaP-652 lung metastasis (L-652-L). Magnifications are 40×.

The acquisition of NED markers by *in vitro* treatment of LNCaP cells with miR-652 mimics raised the possibility that miR-652 may also induce NED *in vivo*. Xenografted primary and metastatic tumors from PC3-652 or LNCaP-652 injected mice were examined for expression of neuroendocrine markers by immunohistochemistry (IHC) (Figure 7). Although the primary tumor derived from PC3-652 was weakly positive compared to PC3-vector control by immunostaining for NED markers, chromogranin A (CGA) and synaptophysin (SYP), the kidney metastasis was negative for these markers. In contrast, while the primary tumor from LNCaP-652 cells was strongly positive for CGA, the lung metastasis exhibited significant positive staining with both CGA and SYP, indicating NED.

## DISCUSSION

In this study, we demonstrated that miR-652 expression levels in the radical prostatectomy specimens are significantly associated with prostate cancer recurrence among men with localized prostate cancer. Further, miR-652 levels could potentially be used to differentiate patients who have a high (or low) risk of developing prostate cancer recurrence, following radical prostatectomy. Although the risk of metastasis was not significantly associated with miR-652 expression, the survival curves of low and high miR-652 expressers diverge, suggesting that the non-significant result reflects a lack of statistical power rather than absence of effect. To our knowledge, this is the first study to identify an association between miR-652 expression levels and biochemical recurrence and metastasis. Analysis of the miR-652 risk score of patients from prostate biopsies at the time of diagnosis may help distinguish patients at low and high risk of recurrence or metastasis, and allow for more nuanced treatment decisions.

Our results suggest that miR-652 is a novel oncogenic miR in aggressive prostate cancer. Previous studies have shown that increased miR-652 expression in non-small cell lung cancer (NSCLC) patient tumors is associated with disease progression and poor survival [43]. MiR-652 expression has been identified along with other miRNAs in diagnostic signature panels associated with gastric, colorectal and breast cancer [44–47]. In NSCLC, overexpression of miR-652-3p promoted proliferation, migration and invasion of NSCLC cells, as well as disease progression and poor survival of NSCLC patients [43]. The overexpression of miR-652 has also been demonstrated in human breast cancer [44], osteosarcoma [48], and rectal cancer [49].

We found that miR-652 increased growth and migration of PC3 and LNCaP prostate cancer cells. MiR-652 overexpression in xenografts derived from either PC3 or LNCaP cells resulted not only in the growth of primary tumors, but also the appearance of metastatic lesions in kidney and lung.

We identified the B” regulatory subunit, PPP2R3A, of the tumor suppressor PP2A, as a potential target of miR-652 using the miRNA target prediction programs miRanda and PicTar. Luciferase assays using the wild type and mutant miR-652 binding sites indicated that PPP2R3A is indeed a direct target of miR-652. The inhibition of both luciferase activity and PPP2R3A expression by miR-652 was blocked by miR-652 antagomir, further verifying that PPP2R3A is indeed a direct target of miR-652 in prostate cancer cells. PP2A is a negative regulator of many signalling pathways promoting cell growth, proliferation, and survival, such as the PI3K/AKT/mTOR, Wnt/β-catenin, c-Myc and the MAP/ERK family of kinases [26–29]. Decreased activity of PP2A is a recurrent observation in many types of cancers, such as colorectal cancer and breast cancer [30, 31]. The cellular inhibitor of PP2A, CIP2A, is an oncogene who’s expression is increased in aggressive forms of prostate cancer [50]. Interestingly, when the frequency of PP2A subunit mutations were examined across 9759 tumor samples, PPP2R3A had the second highest mutational rate (12.16 %) overall, and the highest amongst the 11 B regulatory subunits genes [32].

We show that miR-652 inhibits the expression of PPP2R3A in prostate cancer cells and xenografts (Figures 5C and 6). Suppression of specific regulatory subunits of PP2A is sufficient to induce human cellular transformation since displacing the particular subunit from the rest of the PP2A dimer alters the enzyme’s specific phosphatase activity [51]. In our study, the suppression of PPP2R3A regulatory subunit of PP2A, specifically the PR72 isoform of PPP2R3A, through increased miR-652 expression in prostate cancer cells, correlated with activation of the ERK-1/2, AKT in both PC3 and LNCaP cells, and activation of Wnt signalling pathways, together with inhibition of the p53 and p21 in LNCaP cells.

**Figure 8:**
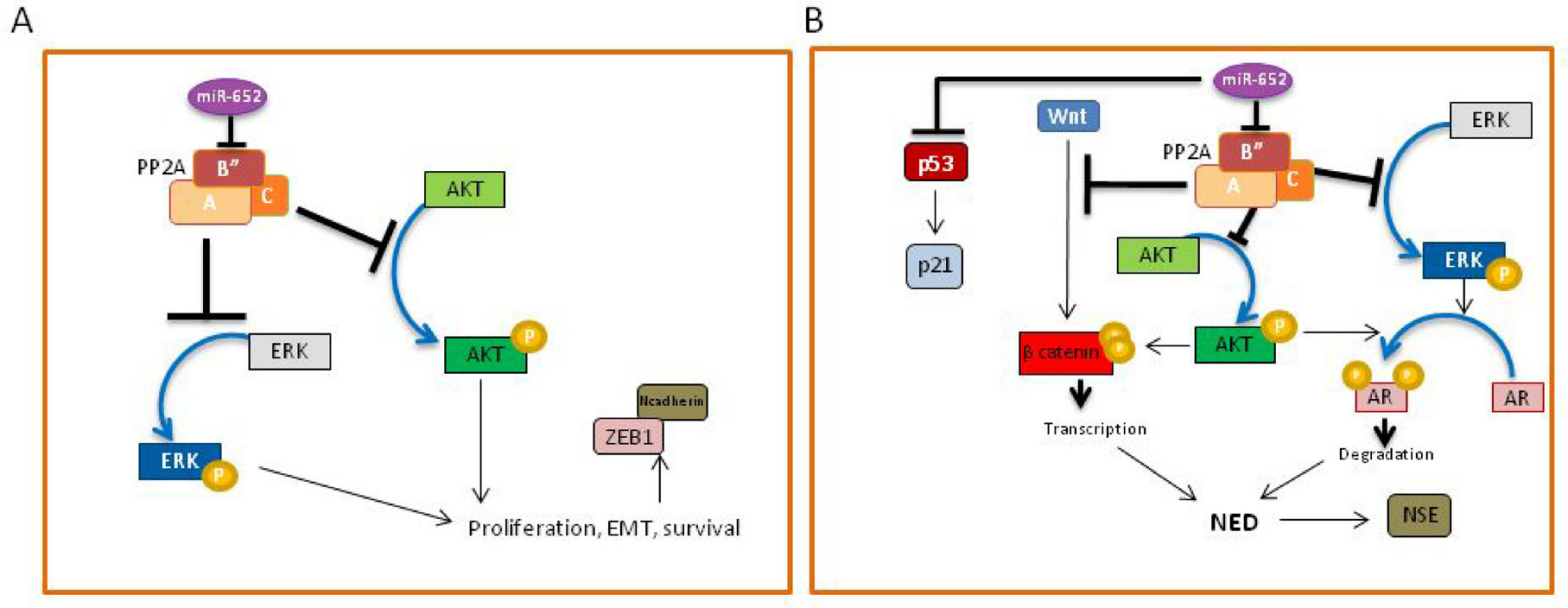
**(A)** Model of miR-652-mediated mechanism of EMT in PC3 cells. The presence of miR-652 results in downregulation of the PPP2R3A regulatory subunit (B”) of PP2A, resulting in inhibition of PP2A activity. This leads to the abrogation of PP2A-mediated inhibition of AKT and ERK kinases, resulting in their phosphorylation and activation. The result is increased cell growth, survival and EMT, with the downregulation of Ecadherin, and upregulation of the mesenchymal markers, N-cadherin and ZEB1. **(B)** Model of miR-652-mediated neuroendocrine differentiation of LNCaP cells. The presence of miR-652 results in downregulation of the PPP2R3A regulatory subunit (B”) of PP2A, resulting in inhibition of PP2A activity. This leads to the abrogation of PP2A-mediated inhibition of AKT and ERK kinases, resulting in their phosphorylation and activation. Activated AKT and ERK are both able to phosphorylate the androgen receptor (AR), resulting in its degradation. PP2A inhibition by miR-652 also releases the Wnt pathway from inhibition, resulting in the phosphorylation (Ser552) and activation of β-catenin, which allows its translocation to the nucleus to impact gene transcription. Activated β-catenin, together with degradation of AR results in transcription of proteins required for neuroendocrine differentiation, specifically, NSE. MiR-652 may also directly inhibit p53, leading to p21 inactivation. Together, this would allow the cells to undergo cell cycle progression.

The mechanism by which miR-652 increased prostate cancer cell aggressiveness and invasiveness varied between PC3 and LNCaP cells. In PC3 cells, we show that miR-652 induced cell morphological changes consistent with mesenchymal cells, coinciding with upregulation of the EMT markers, N-cadherin and ZEB1, and downregulation of E-cadherin. These changes were not observed upon increased miR-652 expression in LNCaP cells. The activation of the PI3K-AKT and ERK-1/2 pathways, both associated with EMT in prostate cancer [52, 53], supported the conclusion that miR-652 induces EMT in PC3 prostate cancer cells (Figure 8A). In contrast, miR-652 overexpression in LNCaP cells resulted in morphological changes indicative of NED. Our results enable us to propose a model of NED, which is initiated by miR-652-mediated abrogation of PP2A activity, via PPP2R3A inhibition (Figure 8B). The PP2A regulatory subunit, PPP2R3A, is directly targeted by miR-652, which would result in diminished PP2A activity. Since the PR72 isoform of PPP2R3A inhibits the Wnt pathway [40], miR-652 overexpression releases this blockage, resulting in activation of the Wnt pathway, as indicated by high levels of phospho-β-catenin (Ser^552^) (Figure 8B). β-catenin, the most downstream event in the Wnt signalling cascade, induces NED when overexpressed in prostate cancer cells [8]. Similarly increased levels of phospho-β-catenin (Ser^552^) are seen in prostate cancer NED models induced by androgen withdrawal [15]. PP2A-mediated inhibition of the MAPK and PI3K pathways is also abrogated upon miR-652 intervention, resulting in activation of ERK-1/2 and AKT (Figure 5). Increased ERK-1/2 phosphorylation strongly correlated with decreased AR, PSA, and increased NSE expression [17]. NED correlates with AKT activation, which in turn regulates AR expression and β-catenin levels [15]. In agreement with this, we observed increased ERK-1/2 and AKT activation, decreased AR expression, and increased activated β-catenin in our model system (Figure 8B). The induction of NED is supported by upregulation of the NED marker, NSE. A similar model of NED has previously been proposed in prostate cancer [15]. However, their initiating event was androgen withdrawal. In our study, LNCaP cells were transfected with miR-652 in the presence of reduced serum, which may be construed as androgen reduced conditions. However, since our negative control mimic did not induce NED even in serum-reduced conditions, we suggest a novel mechanism of NED in prostate cancer that is initiated by miR-652 expression through inhibition of PP2A in LNCaP cells. Interestingly, the microRNA target prediction programs, miRanda and PicTar, also show a miR-652 binding site within certain isoforms of TP53, suggesting that TP53 may be a potential miR-652 target. Since miR-652 expression in LNCaP cells show decreased expression of p53, we cannot rule out the possibility that miR-652 also targets TP53 in our system, which would not only reduce p53 expression, but also inhibit its downstream effector, p21 (Figure 8B). Alternatively, diminished p53 protein expression could have occurred through AKT-mediated increased Mdm2 ubiquitination of p53 [54].

The outcome of NED in LNCaP versus PC3 cells may be a consequence of significantly different characteristics, the most important being that LNCaP cells express the luminal differentiation markers AR and PSA while PC3 cells do not [55]. LNCaP cells are typical of prostatic adenocarcinoma whereas PC3 cells have features typical of prostatic SCNC [55]. Multiple pathways have been shown to induce NED in prostate cancer cells *in vitro*, including androgen deprivation [56, 57], interleukin-6 (IL-6) treatment [58], Wnt pathway activation [8], EGF signaling pathways [59], activation of the cyclic adenosine 3′, 5′-monophosphate (cAMP) signaling pathway [60–62], or ionizing radiation [63, 64]. Thus, the induction of NED in PC3 cells may utilize different signaling pathways compared to LNCaP cells. In our system, PC3 cells were unable to activate β-catenin in response to miR-652, a necessary component of our proposed model of NED by miR-652. Another possible contribution to the divergent phenotypes of miR-652-expressing PC3 and LNCaP cells may involve expression of p53. LNCaP cells contain wild-type *TP53*, whereas PC3 cells have one deleted allele and one frame-shifted producing a stop codon [65]. Data from human prostate tumors indicate that while primary localized tumors usually have wildtype p53, advanced (metastatic and hormone resistant) prostate cancers much more frequently have mutations and/or allelic losses of the p53 gene [66–69]. Mutation of p53 inactivated the IL-8/CXCR2/P53 pathway, resulting in rapid proliferation and aggressive behavior of NE cells and development of SCNC [18], indicating that p53 mutation was a critical event in prostatic SCNC pathogenesis. The inhibition of p53 upon miR-652 expression in LNCaP cells, results in p21 inhibition. PC3 cells lack wild type p53, and in fact have increased p21 expression in response to miR-652, suggesting that p21 in PC3 cells are independent of p53. These differences may contribute to the heterogenic phenotypes that develop in response to miR-652 expression.

PC3 cells exposed to prolonged miR-652 had decreased NSE expression. We showed that miR-652 induces EMT in PC3 cells. The acquisition of chemotherapy and radiotherapy resistance in SCLC is associated with EMT characteristics such as migration and invasion, as well as downregulation of neuroendocrine markers such as NSE [70]. Similar to these findings, the decrease of NSE in PC3 cells exposed to miR-652 in our study indicates that markers of NED are lost during EMT.

Expression of NED-associated markers was not only observed *in vitro*, but also in LNCaP xenografts overexpressing miR-652. Although weak staining of NED markers, CGA and SYP, was observed from PC3-652 derived xenografted primary tumors, the PC3-652 derived kidney metastasis was negative for these markers. In contrast, LNCaP-652-derived xenografts expressed strong CGA staining, and the LNCaP-652 derived lung metastasis was strongly positive for both CGA and SYP expression. These results indicate that not only was miR-652 able to induce NED *in vitro* in LNCaP cells, but also *in vivo*.

In conclusion, our data indicate that miR-652 is a novel oncomiR in prostate cancer cells, inhibiting PP2A activity, through direct targeting of the PP2A regulatory subunit, PPP2R3A. In PC3 prostate cancer cells, miR-652 exposure resulted in EMT. In contrast, miR-652 expression in LNCaP prostate cancer cells induced NED through a pathway initiated by PPP2R3A inhibition, resulting in increased AKT, ERK-1/2 and Wnt activation, loss of AR, with gene transcription via activated β-catenin. Furthermore, the reduction of p53 expression upon miR-652 expression in LNCaP cells may also have contributed to NED. The induction of NED via miR-652 in LNCaP cells was also manifested in metastatic lesions derived from miR-652 overexpressing xenografts. Although rare, neuroendocrine prostate cancer is a lethal subset with poor overall survival. In addition, they are very difficult to treat since they are refractory to hormone therapy due to the lack of AR expression, and resistance to conventional chemotherapy because they do not divide [57]. Mir-652 could serve as a potential biomarker identifying this aggressive subset of prostate tumors early on, leading to more rapid and aggressive treatment. Furthermore, identification of the signaling pathways that facilitate NED may identify future targeting avenues of novel treatments for this aggressive subtype.

## MATERIALS AND METHODS

This study was conducted with the approval of Sunnybrook Health Sciences Centre research ethics board.

### Study cohort

The medical records were systematically reviewed using standardized data entry forms by trained data abstractors and stored within a prostate cancer-specific database. Clinical follow-up consisted of four assessments in the year following surgery, two assessments in the second year and one assessment every year thereafter. At each follow-up, patients had a clinical evaluation, and a serum PSA test. Biochemical recurrence was defined as a PSA increase of at least 0.2 ng/mL on at least two separate consecutive measurements that are at least 3 months apart. Metastasis was defined as lesions within the bone identified on radionuclide bone scan and lymphadenopathy or visceral lesions identified by computed tomography imaging of the abdomen, pelvis and chest. Patients were considered to be at risk from the date of surgery until recurrence or until the date of the last PSA test. Patients who were lost to follow-up were censored at the date of their last PSA test or follow-up.

### Cell lines

Prostate cancer cell-lines were purchased from American Type Culture Collection (ATCC). PC3 prostate cancer cells were maintained in Dulbecco’s Modified Eagle Medium/Nutrient Mixture F-12 (DMEM/F-12) (Wisent) while LNCaP prostate cancer cells were maintained in Roswell Park Memorial Institute (RPMI) media (Wisent), and DU145 prostate cancer cells were maintained in Dulbecco’s modified Eagle’s medium (DMEM) (Wisent), all supplemented with 10% FCS at 37° C in a 5% CO2 atmosphere. HEK293T cells (ATCC) were maintained in DMEM media supplemented with 10% FCS. The normal prostate epithelial cell line, RWPE-1 (ATCC) was maintained in keratinocyte serum-free media (ThermoFisher) supplemented with bovine pituitary extract and recombinant epidermal growth factor.

### Animals

A colony of immunodeficient NOD.CB17 *Prkdcscid*/J mice (Jackson Labs, stock number 001303 (termed NOD/SCID), mice were maintained in-house under aseptic sterile conditions. Mice were given autoclaved food and water. All procedures were approved by the Institutional Research Ethics Board and the Animal Care Committee. Mice were sacrificed upon sign of heavy tumor burden, signs of severe respiratory distress, or weakness and lethargy.

### Reverse transcription and Quantitative Real time PCR

Total RNA was extracted from cell lines using miRNeasy Mini Kit (Qiagen). 1 microgram of total RNA was reverse-transcribed using the miScript II RT kit (Qiagen) for miRNA, or using Superscript™ VILO™ Master Mix for mRNA (ThermoFisher). Quantitative real-time PCR was performed in triplicate using miScript SYBR Green PCR kit (Qiagen) for miRNA on StepOnePlus Real Time PCR system (Life Technologies) with specific primer pairs. The primers for quantification of mature microRNA were purchased from Qiagen. Primers are miR-28 (endogenous control; MS00009254) and miR-652 (MS00010451). Primers for PPP2R3A (PR72) were 5′-CAAGGAAACATCTCTACGAAGG-3′ (forward) and 5′-TCTGTGTCTGCTGAAATGACTT-3′ (reverse); GAPDH were 5′-TGACTCCGACCTTCACCTTC- (forward) and 5′-GCTCTCTGCTCCTCCTGTTC- 3′(reverse).

### Plasmid constructs and establishment of stable and transient transfectants

PCMV-MIR plasmid containing human miR-652 (MI0003667, Origene) or empty vector control (pCMVMIR, Origene) was transfected into PC3 or LNCaP cells using Lipofectamine™ 3000 transfection reagent (Life Technologies). Briefly, for 10 cm^2^ surface area, 5 µg of plasmid DNA was transfected together with 10 µl of Lipofectamine™ 3000 and 10 µl of P3000 reagent. Cells were passaged into fresh media 48 hours post-transfection. Selection reagent, G418 (400 µg/ml), was added 48 hours post-transfection. Once stable cells were established, the GFP+ population was further selected by flow cytometric cell sorting. For transient transfection, PC3 (10^5^/ml) or LNCaP (2 × 10^5^/ml) cells were seeded 24 hours prior to transfection in 6 well plates. Immediately prior to transfection, regular media was replaced with media containing 1% FBS. Transfections were performed using Lipofectamine^®^ RNAiMAX transfection reagent (Thermofisher) according to manufacturer’s recommendations. For prolonged transient transfection, every 3–4 days, media was changed and cells were retransfected with 10 pmol/ml of miR-652 (hsa-miR-652-3p; mirVana™ #MC12699, Thermofisher), or negative control mimic (mirVana™ control mimic #4464058, Thermofisher). For blocking experiments, 20 pmol/ml of anti-hsa-miR-652-3p miScript miRNA inhibitor (Qiagen) were added per well.

### Proliferation assay – thymidine assay

In order to assay growth, stably transfected prostate cancer cells were seeded at 10^4^ cells/well into flat-bottomed 96-well plates (*n* = 6 wells) in media containing 0.5% FBS for 2–6 days. Cells were harvested following a 6-h incubation with 1 µCi/ml [^3^H] thymidine, and scintillation was quantified.

### Migration and invasion assay – trans-well

For migration assay, following incubation for 24 hours in low serum (0.5% FBS) conditions, cells (10^4^ cells/300 µl) were added in low serum (0.5% FBS) containing media to the top chamber of 8 mm-pore-size transwell chambers (Corning Star, Cambridge, MA). The bottom chamber was prepared with 10% FBS as a chemoattractant. Cells were allowed to migrate through the porous membrane for 72 h at 37° C. The cells that had migrated through the membrane and stuck to the lower surface of the membrane were treated with a fixation/staining solution (Diff-Quick) for visualization. For quantification, the cells were counted under a microscope in four randomly selected fields. Invasion assay was similar to migration except that transwell chambers were coated with matrigel (Corning Star). For quantification, cells adhering to bottom side of insert were stained with 0.9% Crystal Violet for 10 minutes, destained with water prior to visualization on the microscope. Stained cells were eluted with extraction solution (10% acetic acid), and absorbance read at 560 nm.

### 3’ UTR construct plasmid design

The sequence of miR-652 and *PPP2R3a* was obtained from NCBI sequence database. The miR-652 binding site in *PPP2R3a*, present in coding region, was obtained from the microRNA databases (microrna.org; pictar.mdc-berlin.de). The *PPP2R3a* sequence was amplified from cDNA sample via nested PCR. Primers (Sigma-Aldrich) were designed for insertion into the pMIR-REPORT™ Luciferase plasmid (Applied Biosystems, Thermo Fisher Scientific), and were as follows: outside primers (forward, 5′- CCTGGAACACCACTCCCACCT -3′; reverse, 5′- CAATATCCATGAAAGCAGTTT -3′); nested primers (forward, 5′- TCAGACTAGTCCTCCAGCCACCTCT -3′; reverse, 5′- TCAGAAGCTTCAATTCTGCTTAGAG -3′). Amplicons and digested pMIR-REPORT™ plasmids were purified using the PureLink™ PCR Micro Kit (Invitrogen). Amplicons were cloned into the SpeI and HindIII sites of the pMIR-REPORT™ plasmid using the Quick Ligation™ kit (New England BioLabs). DH5α competent cells (ThermoFisher Scientific) were used to amplify the constructs. For mutants, custom dsDNA oligonucleotides were designed containing either del3 or del5 deletion mutations in the seed sequence of miR-652 (gBlocks^®^ Gene Fragments, IDT) and cloned as above. Deletions generated novel RE cut sites for NsiI and SspI, respectively, which were used to verify successful cloning into pMIR-REPORT™ construct. All constructs were verified by sequencing at The Centre for Applied Genomics (TCAG, SickKids Hospital, Toronto, ON, Canada) using a pMIR-REPORT™ sequencing primer synthesized with the sequence 5′ – AGGCGATTAAGTTGGGTA – 3′ (IDT). Constructs were extracted (EndoFree^®^ Plasmid Maxi Kit, Qiagen) for downstream Dual-Reporter^®^ Luciferase Assay (Promega).

### Dual-reporter luciferase assay

HEK293T cells were seeded into 24 well plates at 1.5 × 10^5^ cells/well in DMEM + 10% FBS media. Cells were transfected 24 hours later using Lipofectamine^®^ 3000 Transfection Reagent (ThermoFisher Scientific), according to manufacturer`s protocol with 10 ng of either wild-type or mutant pMIR-PPP2R3A reporter plasmid containing firefly luciferase, together with 10 ng of pRL-TK plasmid (Promega, Wisconsin, USA), and 20 pmol of either miR-652 mimic (hsa-miR-652-3p; mirVana™ #MC12699, ThermoFisher Scientific) or (syn-hsa-miR-652-3p miScript mimic; MSY0003322, Qiagen), or scrambled negative control (mirVana™ control mimic #4464058, Thermofisher) were transfected per well. For blocking experiments, 40 pmol of anti-hsa-miR-652-3p miScript miRNA inhibitor (Qiagen) was added per well. Twenty-four hours after transfection, Firefly and Renilla luciferase activity were measured using Dual-Glo Luciferase Reporter Assay System (Promega). The Renilla luciferase activity was normalized to the Firefly luciferase activity. Transfections were done in triplicate, and replicated thrice. A 2-tailed *T*-test was performed. *P*-values < 0.05 were considered significant.

### Western blotting

Cell extracts were prepared by lysis in RIPA buffer in the presence of proteinase and phosphatase inhibitors. The cell lysates were collected by centrifugation at 20,000 g for 15 min at 4° C and protein content in the supernatant was measured using the Bradford protein assay (BioRad, USA). Approximately 10–20 µg of protein were separated on either 8 or 15% SDS-polyacrylamide gels and electroblotted on to nitrocellulose membranes. Membranes were blocked with 5% nonfat dry milk:TBS-T (20 mmol/l Tris-HCl (pH 8.0), 137 mmol/l NaCl and 0.1% Tween 20) or 5% BSA for one hour at RT, and incubated with the primary antibodies overnight at 4°C. After washing with TBS-T, blots were probed with HRP-conjugated secondary antibodies for 1 h at RT. The following monoclonal and polyclonal antibodies were used: anti-PPP2R3A (H-52, Santa Cruz Biotechnology); anti-N-cadherin, anti-E-cadherin (BD Bioscience); anti-phospho-p44/42 MAPK (Erk1/2)(Thr202/Tyr204), anti-p44/42 MAPK (Erk1/2), anti-phospho-Akt(Ser473), anti-Akt, anti-ZEB1 (Cell Signaling); anti-neuron specific enolase (NSE), (Enzo Life Sciences, Exeter, UK); anti-phospho-β catenin (Ser552), (Thermofisher Scientific); mouse anti-β-tubulin (Sigma-Aldrich), and HRP conjugated goat anti-mouse and goat anti-rabbit (Promega).

### Mouse xenografts

Empty vector or miR-652 expressing PC3 (1 × 10^6^ cells/mouse) or LNCaP prostate cancer cells (1 × 10^7^ cells/mouse) were subcutaneously injected together with matrigel (1:1 ratio) into the fat pad of NOD/SCID mice. Five animals for PC3-vector control cell-lines or PC3-miR-652 cell-lines and three animals for LNCaP-vector or LNCaP-miR-652 cell-lines were used. Tumors were measured every 3–4 days. After 8–12 weeks, mice were sacrificed and primary tumors as well as organs were removed for analysis.

### Immunohistochemistry

Paraffin embedded xenografted tumors were used to make 5 μm sections. The slides with tumor sections were deparaffinized in xylene, rehydrated in a graded ethanol series, followed by 10 min incubation in PBS. Slides were then treated with 10 mM citrate buffer (10 mM Sodium Citrate, 0.05% Tween 20, pH 6.0) in a pressure cooker for antigen retrieval. Sections were then sequentially incubated in a humidified chamber in blocking buffer consisting of 10% normal goat serum (Jackson Immunoresearch Laboratories; West Grove, PA) for one hour at room temperature (RT) and primary antibody diluted in blocking buffer overnight at 4° C. Primary antibodies used included rabbit anti-PPP2R3A (Santa Cruz Biotech), rabbit anti-Chromogranin A (Abcam) or mouse anti-Synaptophysin (Enzo Life Sciences). Samples were washed with PBS, and incubated with biotinylated goat anti-mouse or anti-rabbit biotinylated IgG (H+L) secondary antibody (1:200 dilution,Vector; Burlingame, CA) for one hour at RT. Slides were washed again with PBS and incubated with a pre-formed complex of avidin and biotinylated horseradish peroxidase (Vectastain ABC Elite Kit; Vector) for 30 minutes. Immunocomplexes were visualized by the DAB detection system (Vector), and sections were counterstained with hematoxylin (Vector), dehydrated and mounted for examination. Negative controls were serial samples that were processed without primary antibody.

### Statistical analysis

We first examined baseline characteristics of the study population with univariate statistics: Wilcoxon rank sum test for continuous data and Chi-squared test for categorical variables. We then divided the study population in to high and low miR-652 groups based on the median miR-652 level. Univariate analyses using Wilcoxon rank sum test and Chi-squared tests were used to assess the relationship between miR-652 group and accepted prognostic factors.

We performed Kaplan-Meier survival analysis to assess the development of biochemical recurrence and metastasis, stratified by miR-652 group. The log-rank test was used to compare survival outcomes between groups. To examine the independent prognostic effect of miR-652 group, we performed Cox proportional hazard modeling after accounting for patient age at diagnosis, histological grade, pathological stage, PSA category, margin status and lymph node involvement. As there were few events in the metastasis group, this model was over-specified. As a result, we used a more parsimonious model comprising miRNA risk score, histological grade (collapsed to two categories), pathological stage (collapsed to two categories) and PSA category (collapsed to two categories).The proportionality assumption was assessed using time-varying covariates. The assumption was said to hold if no time-varying covariate was significant at *p* < 0.05. Collinearity was assessed using the variance inflation factor (VIF). Collinearity was deemed to be present where the VIF exceeded 2.5. All analyses were performed using SAS 9.4 (SAS Institute Inc., Cary, NC, USA).

## Abbreviations

NED: neuroendocrine differentiation
EMT: epithelial-to-mesenchymal transition
ADT: androgen deprivation therapy
NSCLC: non-small cell lung carcinoma
SCNC: small cell neuroendocrine carcinoma
CRPC: castration resistant prostate cancer
AR: androgen receptor
PI3K: phosphatidylinositol 3-kinase
ERK: extracellular signal-regulated kinase
NSE: neuron specific enolase
CGA: chromogranin A
SYP: synaptophysin
PCR: polymerase chain reaction
PSA: prostate specific antigen
NRC: normalized read count
PP2A: protein phosphatase 2
PPP2R3A: Protein Phosphatase 2 Regulatory Subunit B’’ Alpha
MAPK: mitogen activated protein kinase
CIP2A: Cancerous Inhibitor of PP2A
ZEB1: Zinc finger E-box-binding homeobox 1
FBS: fetal bovine serum
GFP: green fluorescent protein
UTR: untranslated region
RE: restriction enzyme
pRL-TK: thymidine kinase promoter-Renilla luciferase reporter plasmid
BSA: bovine serum albumin
RIPA: Radioimmunoprecipitation Assay Buffer
HRP: horseradish peroxidise
RT: reverse transcription

## References

[1] Komiya A, Suzuki H, Imamoto T, Kamiya N, Nihei N, Naya Y, Ichikawa T, Fuse H (2009). Neuroendocrine differentiation in the progression of prostate cancer. Int J Urol.

[2] Abrahamsson PA (1999). Neuroendocrine differentiation in prostatic carcinoma. Prostate.

[3] Fixemer T, Remberger K, Bonkhoff H (2002). Apoptosis resistance of neuroendocrine phenotypes in prostatic adenocarcinoma. Prostate.

[4] Grobholz R, Griebe M, Sauer CG, Michel MS, Trojan L, Bleyl U (2005). Influence of neuroendocrine tumor cells on proliferation in prostatic carcinoma. Hum Pathol.

[5] Martin-Orozco RM, Almaraz-Pro C, Rodriguez-Ubreva FJ, Cortes MA, Ropero S, Colomer R, Lopez-Ruiz P, Colas B (2007). EGF prevents the neuroendocrine differentiation of LNCaP cells induced by serum deprivation: the modulator role of PI3K/Akt. Neoplasia.

[6] Wu C, Huang J (2007). Phosphatidylinositol 3-kinase-AKT-mammalian target of rapamycin pathway is essential for neuroendocrine differentiation of prostate cancer. J Biol Chem.

[7] Uysal-Onganer P, Kawano Y, Caro M, Walker MM, Diez S, Darrington RS, Waxman J, Kypta RM (2010). Wnt-11 promotes neuroendocrine-like differentiation, survival and migration of prostate cancer cells. Mol Cancer.

[8] Yang X, Chen MW, Terry S, Vacherot F, Chopin DK, Bemis DL, Kitajewski J, Benson MC, Guo Y, Buttyan R (2005). A human- and male-specific protocadherin that acts through the wnt signaling pathway to induce neuroendocrine transdifferentiation of prostate cancer cells. Cancer Res.

[9] Zhu H, Mazor M, Kawano Y, Walker MM, Leung HY, Armstrong K, Waxman J, Kypta RM (2004). Analysis of Wnt gene expression in prostate cancer: mutual inhibition by WNT11 and the androgen receptor. Cancer Res.

[10] Strack S (2002). Overexpression of the protein phosphatase 2A regulatory subunit Bgamma promotes neuronal differentiation by activating the MAP kinase (MAPK) cascade. J Biol Chem.

[11] Gioeli D, Mandell JW, Petroni GR, Frierson HF, Weber MJ (1999). Activation of mitogen-activated protein kinase associated with prostate cancer progression. Cancer Res.

[12] Kinkade CW, Castillo-Martin M, Puzio-Kuter A, Yan J, Foster TH, Gao H, Sun Y, Ouyang X, Gerald WL, Cordon-Cardo C, Abate-Shen C (2008). Targeting AKT/mTOR and ERK MAPK signaling inhibits hormone-refractory prostate cancer in a preclinical mouse model. J Clin Invest.

[13] Royuela M, Arenas MI, Bethencourt FR, Sanchez-Chapado M, Fraile B, Paniagua R (2002). Regulation of proliferation/apoptosis equilibrium by mitogen-activated protein kinases in normal, hyperplastic, and carcinomatous human prostate. Hum Pathol.

[14] Uzgare AR, Kaplan PJ, Greenberg NM (2003). Differential expression and/or activation of P38MAPK, erk1/2, and jnk during the initiation and progression of prostate cancer. Prostate.

[15] Ciarlo M, Benelli R, Barbieri O, Minghelli S, Barboro P, Balbi C, Ferrari N (2012). Regulation of neuroendocrine differentiation by AKT/hnRNPK/AR/beta-catenin signaling in prostate cancer cells. Int J Cancer.

[16] Krijnen JL, Janssen PJ, Ruizeveld de Winter JA, van Krimpen H, Schröder FH, van der Kwast TH (1993). Do neuroendocrine cells in human prostate cancer express androgen receptor?. Histochemistry.

[17] Hong SK, Kim JH, Lin MF, Park JI (2011). The Raf/MEK/extracellular signal-regulated kinase 1/2 pathway can mediate growth inhibitory and differentiation signaling via androgen receptor downregulation in prostate cancer cells. Exp Cell Res.

[18] Chen H, Sun Y, Wu C, Magyar CE, Li X, Cheng L, Yao JL, Shen S, Osunkoya AO, Liang C, Huang J (2012). Pathogenesis of prostatic small cell carcinoma involves the inactivation of the P53 pathway. Endocr Relat Cancer.

[19] Huang J, Yao JL, Zhang L, Bourne PA, Quinn AM, di Sant’Agnese PA, Reeder JE (2005). Differential expression of interleukin-8 and its receptors in the neuroendocrine and non-neuroendocrine compartments of prostate cancer. Am J Pathol.

[20] Hagood PG, Johnson FE, Bedrossian CW, Silverberg AB (1991). Small cell carcinoma of the prostate. Cancer.

[21] Liang H, Studach L, Hullinger RL, Xie J, Andrisani OM (2014). Down-regulation of RE-1 silencing transcription factor (REST) in advanced prostate cancer by hypoxia-induced miR-106b∼25. Exp Cell Res.

[22] Zheng C, Yinghao S, Li J (2012). MiR-221 expression affects invasion potential of human prostate carcinoma cell lines by targeting DVL2. Med Oncol.

[23] Jiao L, Deng Z, Xu C, Yu Y, Li Y, Yang C, Chen J, Liu Z, Huang G, Li LC, Sun Y (2014). miR-663 induces castration-resistant prostate cancer transformation and predicts clinical recurrence. J Cell Physiol.

[24] Nam RK, Amemiya Y, Benatar T, Wallis CJ, Stojcic-Bendavid J, Bacopulos S, Bacopulos S, Sherman C, Sugar L, Naeim M, Yang W, Zhang A, Klotz LH (2015). Identification and Validation of a Five MicroRNA Signature Predictive of Prostate Cancer Recurrence and Metastasis: A Cohort Study. J Cancer.

[25] Eichhorn PJ, Creyghton MP, Bernards R (2009). Protein phosphatase 2A regulatory subunits and cancer. Biochim Biophys Acta.

[26] Hahn K, Miranda M, Francis VA, Vendrell J, Zorzano A, Teleman AA (2010). PP2A regulatory subunit PP2A-B’ counteracts S6K phosphorylation. Cell Metab.

[27] Sablina AA, Hector M, Colpaert N, Hahn WC (2010). Identification of PP2A complexes and pathways involved in cell transformation. Cancer Res.

[28] Silverstein AM, Barrow CA, Davis AJ, Mumby MC (2002). Actions of PP2A on the MAP kinase pathway and apoptosis are mediated by distinct regulatory subunits. Proc Natl Acad Sci U S A.

[29] Zhang Q, Claret FX (2012). Phosphatases: the new brakes for cancer development?. Enzyme Res.

[30] Baldacchino S, Saliba C, Petroni V, Fenech AG, Borg N, Grech G (2014). Deregulation of the phosphatase, PP2A is a common event in breast cancer, predicting sensitivity to FTY720. EPMA J.

[31] Cristobal I, Manso R, Rincon R, Carames C, Senin C, Borrero A, Martinez-Useros J, Rodriguez M, Zazo S, Aguilera O, Madoz-Gurpide J, Rojo F, Garcia-Foncillas J (2014). PP2A inhibition is a common event in colorectal cancer and its restoration using FTY720 shows promising therapeutic potential. Mol Cancer Ther.

[32] Sangodkar J, Farrington CC, McClinch K, Galsky MD, Kastrinsky DB, Narla G (2016). All roads lead to PP2A: exploiting the therapeutic potential of this phosphatase. FEBS J.

[33] Nam RK, Benatar T, Wallis CJ, Amemiya Y, Yang W, Garbens A, Naeim M, Sherman C, Sugar L, Seth A (2016). MiR-301a regulates E-cadherin expression and is predictive of prostate cancer recurrence. Prostate.

[34] Janssens V, Goris J (2001). Protein phosphatase 2A: a highly regulated family of serine/threonine phosphatases implicated in cell growth and signalling. Biochem J.

[35] Janssens V, Goris J, Van HC (2005). PP2A: the expected tumor suppressor. Curr Opin Genet Dev.

[36] Bhardwaj A, Singh S, Srivastava SK, Honkanen RE, Reed E, Singh AP (2011). Modulation of protein phosphatase 2A activity alters androgen-independent growth of prostate cancer cells: therapeutic implications. Mol Cancer Ther.

[37] el-Deiry WS, Tokino T, Velculescu VE, Levy DB, Parsons R, Trent JM, Lin D, Mercer WE, Kinzler KW, Vogelstein B (1993). WAF1, a potential mediator of p53 tumor suppression. Cell.

[38] Kaul R, Mukherjee S, Ahmed F, Bhat MK, Chhipa R, Galande S, Chattopadhyay S (2003). Direct interaction with and activation of p53 by SMAR1 retards cell-cycle progression at G2/M phase and delays tumor growth in mice. Int J Cancer.

[39] Suzuki H, Ito R, Ikeda K, Tamura TA (2012). TATA-binding protein (TBP)-like protein is required for p53-dependent transcriptional activation of upstream promoter of p21Waf1/Cip1 gene. J Biol Chem.

[40] Creyghton MP, Roel G, Eichhorn PJ, Hijmans EM, Maurer I, Destree O, Bernards R (2005). PR72, a novel regulator of Wnt signaling required for Naked cuticle function. Genes Dev.

[41] Igawa T, Lin FF, Rao P, Lin MF (2003). Suppression of LNCaP prostate cancer xenograft tumors by a prostate-specific protein tyrosine phosphatase, prostatic acid phosphatase. Prostate.

[42] Okamoto R, Delansorne R, Wakimoto N, Doan NB, Akagi T, Shen M, Ho QH, Said JW, Koeffler HP (2012). Inecalcitol, an analog of 1alpha, 25(OH) D, induces growth arrest of androgen-dependent prostate cancer cells. Int J Cancer.

[43] Yang W, Zhou C, Luo M, Shi X, Li Y, Sun Z, Zhou F, Chen Z, He J (2016). MiR-652-3p is upregulated in non-small cell lung cancer and promotes proliferation and metastasis by directly targeting Lgl1. Oncotarget.

[44] Cuk K, Zucknick M, Madhavan D, Schott S, Golatta M, Heil J, Marme F, Turchinovich A, Sinn P, Sohn C, Junkermann H, Schneeweiss A, Burwinkel B (2013). Plasma microRNA panel for minimally invasive detection of breast cancer. PLoS One.

[45] Kleivi SK, Bottai G, Naume B, Burwinkel B, Calin GA, Borresen-Dale AL, Santarpia L (2015). A serum microRNA signature predicts tumor relapse and survival in triple-negative breast cancer patients. Clin Cancer Res.

[46] Liu R, Zhang C, Hu Z, Li G, Wang C, Yang C, Huang D, Chen X, Zhang H, Zhuang R, Deng T, Liu H, Yin J (2011). A five-microRNA signature identified from genome-wide serum microRNA expression profiling serves as a fingerprint for gastric cancer diagnosis. Eur J Cancer.

[47] Shin VY, Ng EK, Chan VW, Kwong A, Chu KM (2015). A three-miRNA signature as promising non-invasive diagnostic marker for gastric cancer. Mol Cancer.

[48] Lulla RR, Costa FF, Bischof JM, Chou PM, de F Bonaldo M, Vanin EF, Soares MB (2011). Identification of Differentially Expressed MicroRNAs in Osteosarcoma. Sarcoma.

[49] Gaedcke J, Grade M, Camps J, Sokilde R, Kaczkowski B, Schetter AJ, Difilippantonio MJ, Harris CC, Ghadimi BM, Moller S, Beissbarth T, Ried T, Litman T (2012). The rectal cancer microRNAome—microRNA expression in rectal cancer and matched normal mucosa. Clin Cancer Res.

[50] Vaarala MH, Vaisanen MR, Ristimaki A (2010). CIP2A expression is increased in prostate cancer. J Exp Clin Cancer Res.

[51] Chen W, Possemato R, Campbell KT, Plattner CA, Pallas DC, Hahn WC (2004). Identification of specific PP2A complexes involved in human cell transformation. Cancer Cell.

[52] Bitting RL, Armstrong AJ (2013). Targeting the PI3K/Akt/mTOR pathway in castration-resistant prostate cancer. Endocr Relat Cancer.

[53] Mulholland DJ, Kobayashi N, Ruscetti M, Zhi A, Tran LM, Huang J, Gleave M, Wu H (2012). Pten loss and RAS/MAPK activation cooperate to promote EMT and metastasis initiated from prostate cancer stem/progenitor cells. Cancer Res.

[54] Ogawara Y, Kishishita S, Obata T, Isazawa Y, Suzuki T, Tanaka K, Masuyama N, Gotoh Y (2002). Akt enhances Mdm2-mediated ubiquitination and degradation of p53. J Biol Chem.

[55] Tai S, Sun Y, Squires JM, Zhang H, Oh WK, Liang CZ, Huang J (2011). PC3 is a cell line characteristic of prostatic small cell carcinoma. Prostate.

[56] Ismail AH, Altaweel W, Chevalier S, Kassouf W, Aprikian AG (2004). Expression of vascular endothelial growth factor-A in human lymph node metastases of prostate cancer. Can J Urol.

[57] Yuan TC, Veeramani S, Lin MF (2007). Neuroendocrine-like prostate cancer cells: neuroendocrine transdifferentiation of prostate adenocarcinoma cells. Endocr Relat Cancer.

[58] Deeble PD, Murphy DJ, Parsons SJ, Cox ME (2001). Interleukin-6- and cyclic AMP-mediated signaling potentiates neuroendocrine differentiation of LNCaP prostate tumor cells. Mol Cell Biol.

[59] Cortés MA, Cariaga-Martinez AE, Lobo MV, Martín Orozco RM, Motiño O, Rodríguez-Ubreva FJ, Angulo J, López-Ruiz P, Colás B (2012). EGF promotes neuroendocrine-like differentiation of prostate cancer cells in the presence of LY294002 through increased ErbB2 expression independent of the phosphatidylinositol 3-kinase-AKT pathway. Carcinogenesis.

[60] Cantile M, Kisslinger A, Cindolo L, Schiavo G, D’Anto V, Franco R, Altieri V, Gallo A, Villacci A, Tramontano D, Cillo C (2005). cAMP induced modifications of HOX D gene expression in prostate cells allow the identification of a chromosomal area involved *in vivo* with neuroendocrine differentiation of human advanced prostate cancers. J Cell Physiol.

[61] Cox ME, Deeble PD, Bissonette EA, Parsons SJ (2000). Activated 3′,5′-cyclic AMP-dependent protein kinase is sufficient to induce neuroendocrine-like differentiation of the LNCaP prostate tumor cell line. J Biol Chem.

[62] Zelivianski S, Verni M, Moore C, Kondrikov D, Taylor R, Lin MF (2001). Multipathways for transdifferentiation of human prostate cancer cells into neuroendocrine-like phenotype. Biochim Biophys Acta.

[63] Deng X, Liu H, Huang J, Cheng L, Keller ET, Parsons SJ, Hu CD (2008). Ionizing radiation induces prostate cancer neuroendocrine differentiation through interplay of CREB and ATF2: implications for disease progression. Cancer Res.

[64] Deng X, Elzey BD, Poulson JM, Morrison WB, Ko SC, Hahn NM, Ratliff TL, Hu CD (2011). Ionizing radiation induces neuroendocrine differentiation of prostate cancer cells *in vitro*, *in vivo* and in prostate cancer patients. Am J Cancer Res.

[65] Chappell WH, Lehmann BD, Terrian DM, Abrams SL, Steelman LS, McCubrey JA (2012). p53 expression controls prostate cancer sensitivity to chemotherapy and the MDM2 inhibitor Nutlin-3. Cell Cycle.

[66] Apakama I, Robinson MC, Walter NM, Charlton RG, Royds JA, Fuller CE, Neal DE, Hamdy FC (1996). bcl-2 overexpression combined with p53 protein accumulation correlates with hormone-refractory prostate cancer. Br J Cancer.

[67] Eastham JA, Stapleton AM, Gousse AE, Timme TL, Yang G, Slawin KM, Wheeler TM, Scardino PT, Thompson TC (1995). Association of p53 mutations with metastatic prostate cancer. Clin Cancer Res.

[68] Meyers FJ, Gumerlock PH, Chi SG, Borchers H, Deitch AD, deVere White RW (1998). Very frequent p53 mutations in metastatic prostate carcinoma and in matched primary tumors. Cancer.

[69] Navone NM, Labate ME, Troncoso P, Pisters LL, Conti CJ, von Eschenbach AC, Logothetis CJ (1999). p53 mutations in prostate cancer bone metastases suggest that selected p53 mutants in the primary site define foci with metastatic potential. J Urol.

[70] Krohn A, Ahrens T, Yalcin A, Plones T, Wehrle J, Taromi S, Wollner S, Follo M, Brabletz T, Mani SA, Claus R, Hackanson B, Burger M (2014). Tumor cell heterogeneity in Small Cell Lung Cancer (SCLC): phenotypical and functional differences associated with Epithelial-Mesenchymal Transition (EMT) and DNA methylation changes. PLoS One.

